# Impact on Clinical Management of After-Hours Emergent or Urgent Breast Ultrasonography in Patients with Clinically Suspected Breast Abscesses

**DOI:** 10.3390/diagnostics8010017

**Published:** 2018-02-23

**Authors:** Tanya W. Moseley, Ashley Stanley, Wei Wei, Jay R. Parikh

**Affiliations:** 1 Section of Breast Imaging, Department of Diagnostic Radiology, The University of Texas MD Anderson Cancer Center, 1155 Pressler St., Unit 1350, Houston, TX 77030, USA; Ashley.Stanley@gmail.com (A.S.); jrparikh@mdanderson.org (J.R.P.); 2 Radiology Partners, CHI St. Luke’s Way, The Woodlands, Houston, TX 77384, USA; 3 Department of Biostatistics, The University of Texas MD Anderson Cancer Center, 1515 Holcombe Blvd, Houston, TX 77030, USA; wwei@mdanderson.org

**Keywords:** breast ultrasound, breast abscess, after-hours imaging, urgent or emergent imaging

## Abstract

Newly diagnosed breast abscesses are generally treated as a medical emergency that may necessitate immediate interventional treatment. At our institution, there is no in-house after-hours coverage for breast ultrasonography. We could find no peer-reviewed studies on the cost-effectiveness or clinical management impact of on-call ultrasound technologist coverage for imaging of breast abscesses. The purposes of this study were to determine the incidence of breast abscess in patients with clinical findings highly suggestive of abscess, identify clinical factors associated with breast abscess in such patients, and determine the impact of after-hours emergent or urgent breast ultrasonography on the clinical management of breast abscesses in both outpatients and inpatients. We retrospectively reviewed 100 after-hours breast ultrasound studies performed at our tertiary care center from 2011 to 2015 for evaluation of a suspected breast abscess. Only 26% of our patients with clinically suspected abscess ultimately had a confirmed abscess. Factors associated with breast abscess were a palpable abnormality and a history of breast surgery within the eight weeks before presentation. After-hours diagnosis of an abscess was associated with after-hours clinical intervention. Of the 74 patients in whom after-hours ultrasound imaging showed no evidence of abscess, only three patients underwent after-hours drainage. Our findings support overnight and weekend breast ultrasound coverage in large tertiary care centers.

## 1. Introduction

Breast abscesses are collections of infected fluid or pus within the breast and occur primarily as a complication of bacterial infectious mastitis [1,2]. When the infected area of the breast becomes walled off, a breast abscess occurs [1]. Although there are no universally accepted guidelines for the management of newly diagnosed breast abscesses [3,4], they are generally treated as a medical emergency that may require immediate interventional treatment [5]. While findings on physical examination such as erythema, tenderness, palpable mass, and fever are considered fairly sensitive in the detection of breast infections, ultrasonography remains the imaging modality of choice [1] to urgently diagnose a breast abscess as it is considered beneficial to triage patients in the emergency department and to influence management in the inpatient setting. Every female with inflammatory breast symptoms suggestive of an abscess that fails to resolve with conservative management should receive ultrasound evaluation [2].

At our large tertiary care center, there is no in-house after-hours coverage for breast ultrasonography. Therefore, for emergent or urgent evaluation of a clinically suspected breast abscess on the weekend or on weeknights between the hours of 5:00 p.m. and 7:00 a.m., the on-call radiologist and technologist must return to the hospital to perform and interpret the examination. 

In the United States, reimbursement for imaging services is shifting from volume-based reimbursement to value-based reimbursement [6]. In response to this shift, the American College of Radiology is advocating the Imaging 3.0 cultural transformation, whereby radiologists demonstrate value beyond image interpretation to patients, referring clinicians, and their institutions [7]. The performance of after-hours breast ultrasonography potentially aligns with this proposed culture shift, as long as there is demonstrable value in performing ultrasonography. However, whereas the cost-effectiveness of 24-h ultrasound technologist coverage for emergent general/body imaging has been well documented [8], we could find no peer-reviewed studies regarding the cost-effectiveness or impact on clinical management of on-call radiologist and ultrasound technologist coverage for imaging of breast abscesses.

The purpose of the present study was threefold: (i) to determine the incidence of breast abscess in patients with clinical findings highly suggestive of abscess, (ii) to identify clinical factors associated with breast abscess in such patients at our institution, and (iii) to determine the impact of after-hours emergent or urgent breast ultrasonography on the clinical management of breast abscesses in both outpatients and inpatients.

## 2. Materials and Methods

### 2.1. Case Identification

Our institutional review board approved this HIPAA (Health Insurance Portability and Accountability Act)-compliant retrospective cohort study and waived the requirement for informed consent. Our institution’s radiology database was queried to identify all patients seen in our hospital, our emergency department, or one of our outpatient clinics who underwent after-hours emergent or urgent ultrasonography in our radiology department for a clinically suspected breast abscess during the period from 1 January 2011 through 31 December 2015. After hours was defined as before 7 a.m. or after 5 p.m., Monday through Friday, and any time Saturday or Sunday. 

Patients were excluded if they eventually underwent surgery or other interventional treatment for a breast condition other than a breast abscess (i.e., surgery for breast cancer), if they underwent after-hours breast imaging for an indication other than a suspected breast abscess, or if they underwent non-urgent evening ultrasound evaluation of a suspected breast abscess because of a delay in the clinic or a late appointment for non-urgent imaging. Because our patient population is predominantly women well outside of the breast-feeding period, we focused on non-puerperal patients presenting with clinically suspected abscesses. Breast-feeding women were excluded because breast abscesses in breast-feeding women differ from breast abscesses in non-breast-feeding women in terms of presentation, risk factors, and recommended management [2]. A total of 100 patients met the inclusion and exclusion criteria, and their medical records and radiology images were reviewed. 

### 2.2. Ultrasonography and Interpretation of Ultrasound Studies

The studies were performed on Philips IU22 (Philips Ultrasound, Bothell, WA, USA), Philips EPIC, and Siemens Antares machines (Siemens Ultrasound, Malvern, PA, USA) by dedicated academic breast ultrasound technologists and dedicated academic breast radiologists. All breast ultrasound studies were independently reviewed for this study by two blinded readers. The participating readers were a breast-imaging fellow (Ashley Stanley) and two fellowship-trained breast imaging faculty members (Jay R. Parikh and Tanya W. Moseley) with 20 and 21 years of breast imaging experience, respectively. A breast abscess was defined at the time of ultrasonography as a walled-off fluid collection and/or inflammatory mass. Criteria used to diagnose abscess were hypoechoic collection of variable size and shape, with or without possible debris and posterior enhancement, eccentrically thickened walls, markedly increased color Doppler flow in the walls and surrounding tissue, and lack of internal color Doppler flow [1]. Final review required two-reader agreement. If there was disagreement, consensus was reached after discussion between the 2 readers.

### 2.3. Data Collection and Interpretation

For each patient, the following data were extracted from the medical records: status at time of presentation with symptoms suggestive of abscess (emergency department patient, inpatient, or outpatient), indication for ultrasonography (e.g., fever, redness, swelling, focal pain, and/or palpable abnormality), age at time of ultrasonography for suspected breast abscess; history of breast abscess, history of breast cancer, history of breast surgery; history of smoking, history of diabetes, immunocompromised status (yes or no), time of initial presentation with symptoms suggestive of breast abscess, time of request for ultrasonography, time ultrasound examination started, imaging findings (positive for abscess vs negative for abscess), management of confirmed breast abscesses by ultrasonography (whether or not drainage was performed), and time of drainage (whether or not it was performed after hours).

Data were analyzed to determine the incidence of breast abscesses in the study population; risk factors for development of breast abscess; and time from imaging to drainage. 

Immunocompromised patients were defined as those with current steroid use, known HIV/AIDS, or current chemotherapy. Emergency department patients were defined as patients who underwent ultrasonography for suspected breast abscess after presenting to the emergency department on their own initiative or after a telephone consultation during which their ordering provider advised them to go to the emergency department. Inpatients were defined as patients who underwent ultrasonography for suspected breast abscess after hospital admission. Outpatients were defined as patients who underwent ultrasonography for suspected breast abscess following an outpatient clinic visit without hospital admission.

### 2.4. Statistical Analysis

Patient characteristics and symptom status were summarized using frequencies and percentages overall and by abscess status. Patient age and time from after-hours imaging to drainage were summarized using mean, standard deviation, and range. Fisher’s exact test was used to assess association between clinical factors and abscess status. The Wilcoxon rank-sum test was used to compare age and time from imaging to interventional treatment between abscess groups. All tests were two-sided, and *p* values of 0.05 or less were considered statistically significant. Statistical analysis was carried out using SAS, version 9.4 (SAS Institute, Cary, NC, USA).

## 3. Results

Demographic and clinical characteristics of the 100 study subjects are presented in Table 1.

### 3.1. Incidence and Treatment of Abscesses

Of the 100 patients, 26 (26%) had ultrasound findings suggestive of a breast abscess. The incidence of breast abscess did not differ significantly by patient location at presentation with symptoms suggestive of breast abscess: 6 of the 26 (23%) emergency department patients, 12 of the 45 (27%) inpatients, and 8 of the 29 (28%) outpatients had ultrasound findings suggestive of a breast abscess.

Of these 26 patients, 22 (85%) underwent interventional treatment, seven after hours (immediately after imaging) and 15 during normal business hours, and four did not undergo any interventional treatment. Of the 74 patients who had no ultrasound evidence of an abscess, 18 patients (24%) underwent interventional treatment, three after hours (immediately after imaging) and 15 during normal business hours, and 56 did not undergo any interventional treatment. Thus, a total of 40 patients underwent an interventional treatment at any time point (10 surgery; 30 percutaneous drainage), and 18 of these patients (45%) had no evidence of abscess on ultrasonography. Ten patients (10%) underwent an interventional treatment after hours, and three of these patients (30%) had no evidence of abscess on ultrasonography.

Two patients underwent surgical management for suspected implant infection in the absence of ultrasound evidence of abscess. In two patients, interventions were attempted but no drainable fluid collection was present at the time of intervention. Finally, one patient who underwent intervention during normal business hours was diagnosed with malignancy.

### 3.2. Risk Factors for Abscess

Of the demographic and clinical factors examined, only a palpable abnormality and a history of breast surgery within 8 weeks prior to ultrasonography for suspected breast abscess were significantly associated with abscess (Table 1). Sixty patients (60%) had undergone breast surgery within the prior 8 weeks. Ultrasonography showed evidence of an abscess in 35% (21/60) of the patients reporting a history of recent surgery compared to 12.5% (5/40) of the patients without a recent history of surgery (*p* = 0.02). Nine patients (9%) demonstrated a palpable abnormality on physical examination at the time of presentation. Ultrasonography showed evidence of an abscess in 56% (5/9) of the patients with a palpable abnormality compared to 23% (21/90) of the patients without a palpable abnormality (*p* = 0.05).

Median age did not differ between the 26 patients with breast abscesses (54.5 years (range 13–71 years)) and the 74 patients without breast abscess (50.5 years (range 31–82 years).

### 3.3. Impact of After-Hours Breast Ultrasonography on Clinical Management

After-hours diagnosis by ultrasonography of a breast abscess was significantly associated with after-hours intervention. Patients with ultrasound confirmation of a breast abscess were more likely to undergo after-hours intervention than were patients without ultrasound confirmation of an abscess (*p* = 0.003; Table 1). Twenty-seven percent (7/26) of the patients with a confirmed abscess underwent after-hours drainage, and 96% (71/74) of the patients without a confirmed abscess did not undergo after-hours drainage.

Out of the 26 patients in which ultrasound demonstrated suspicion of an abscess, 22 received an intervention at some time point (seven after-hours and 15 during normal hours). Four patients did not receive intervention. Of the 74 patients in which after-hours ultrasound did not demonstrate evidence of an abscess, 18 received an intervention at some time point (three after-hours and 15 during normal hours). Eight-five percent (22/26) of the patients with a confirmed abscess underwent drainage at some time, and 76% (56/74) of the patients without a confirmed abscess did not undergo drainage at any time. 

## 4. Discussion

In this cohort of 100 patients who underwent after-hours emergent or urgent ultrasonography for evaluation of clinically suspected breast abscesses, we found that the incidence of breast abscess was 26%. Factors associated with breast abscess were palpable abnormality and history of breast surgery within eight weeks prior to ultrasonography. 

Previously reported incidences of breast abscess range from 40 to 65% [9]. In contrast, the incidence in our study, which was limited to patients with clinically suspected breast abscesses in patients evaluated at a tertiary care center, was only 26%. This difference is most likely related to differences in case mix and patient populations.

Previously reported risk factors for nonpuerperal breast abscess include smoking, recent surgical intervention, previous abscess, and possibly diabetes mellitus [10,11]. Gollapalli et al. [12] demonstrated that cigarette smoking, obesity, diabetes mellitus, and nipple piercing were significant risk factors for the development of primary breast abscesses, while smoking, older age, and recent surgical treatment were associated with the development of recurrent breast abscesses [10]. In agreement with these previous reports, we found an association between recent surgery and breast abscess. However, in contrast with previous reports, we found no association between age, smoking history, and personal history of diabetes mellitus and breast abscess. Finally, we also identified palpable breast abnormality as a risk factor for confirmed breast abscess. Seemingly reliable physical examination findings, including fever, erythema, and swelling, were not significantly associated with a breast abscess in our study. These differences may be attributable to differences in patient populations.

Traditionally, surgical incision and drainage requiring general anesthesia was the gold standard for treatment of a breast abscess [2]. However, as increasing weight has been placed on the role of percutaneous drainage and ultrasound follow-up, the roles of the breast radiologist and interventional radiologist in the management of breast abscesses have increased, and surgical management is no longer recommended as the mainstay of treatment [3]. Motivated by a lack of reliable studies on management of breast abscesses, Trop et al. conducted a comprehensive review [2] synthesizing data spanning 20 years and established an evidence-based algorithm for the management of breast abscesses. In addition to recommending that antibiotic treatment always accompany intervention, the authors concluded that ultrasound evaluation remained invaluable in the diagnosis, treatment, and follow-up of breast abscesses as ultrasound evaluation is relatively low-cost, easily accessible, and can guide percutaneous intervention under local anesthesia, which is associated with lower complication rates than open surgical intervention.

At our institution, because there is no after-hours in-house coverage for breast ultrasonography, emergent and urgent requests for evaluation of clinically suspected breast abscess require the on-call breast radiologist and technologist to return to the hospital to perform and interpret the examination. Our study showed that after-hours breast ultrasonography had an impact on clinical management of abscesses. Patients with after-hours ultrasound confirmation of an abscess were significantly more likely to undergo after-hours intervention than were patients without a confirmed abscess. In addition to allowing immediate intervention in the 26% of patients with confirmed breast abscess, ultrasonography relieved anxiety for the 74% of patients without breast abscess and the physicians caring for them. Patients with after-hours ultrasound findings not suggestive of an abscess avoided after-hours intervention 96% of the time. The radiologists quickly provided a directive ultrasound evaluation that either enabled for rapid treatment or relieved the patient’s anxiety and the physician’s clinical concern. 

Currently, a dynamic transition is under way in the United States from volume-based reimbursement in health care to value-based, patient-focused reimbursement. In response, the American College of Radiology is advocating the Imaging 3.0 cultural transformation in radiology, in which radiologists should step away from the PACS workstations and demonstrate their value to patients, clinicians, and society [7]. Breast imaging is the paradigm in radiology for value-based care [13], and breast imaging is considered the face of Imaging 3.0 [14]. Breast imagers interact with patients daily and participate in activities such as multidisciplinary tumor boards [15] to demonstrate value. Our study shows that clinical management of breast abscesses is impacted by radiologists performing breast ultrasonography after hours. This involvement of breast imagers in the management of breast abscesses is yet another way for breast imagers to potentially demonstrate value, in line with the American College of Radiology’s Imaging 3.0 initiative, and be role models in radiology.

While we accept the necessity of ultrasonography in the management of clinically suspected breast abscess, we also acknowledge the need to establish the relative urgency of after-hours evaluation of suspected breast abscess. As our study demonstrated only a 26% incidence of breast abscess in our population, the majority of these urgent examinations were negative for breast abscess. Additionally, the diagnosis of a breast abscess was not always associated with receipt of after-hours intervention. Only seven of the 26 patients with after-hours ultrasound confirmation of a breast abscess had an immediate after-hours intervention. When a breast abscess was confirmed via ultrasonography, most often patients first received conservative therapy with antibiotics and then underwent planned intervention during normal business hours [2]. 

Our study had limitations. Our study was performed at a tertiary care cancer center with complex patients. Nearly all of our patients were currently being treated for breast cancer, had a history of breast cancer, and/or had a history of a recent breast procedure. The case mix is likely different from that at other tertiary care centers. These results may not extrapolate to smaller community hospitals where patients have less complicated conditions and the incidence of breast abscesses may be lower. To our knowledge, this is the first study of this type to have been carried out in a large tertiary care center, and future research and corroborating studies are needed before this practice model be implemented at other facilities. At our institution, we had a change from one electronic medical record to a new one since the studied time period, and some of the data were missing from the electronic medical records.

At our own facility, this study supports our practice model for after-hours breast ultrasound coverage by a radiologist and technologist. Specifically, the data show that this practice model helps triage patients into those potentially benefitting from intervention. Since clinical management is impacted, our clinicians and radiologists continue to support the coverage. Future studies are encouraged to see if these results are replicated in other breast centers and to evaluate the cost-effectiveness of after-hours ultrasound coverage for breast abscess management.

## Figures and Tables

**Table 1:** Patient characteristics overall and by abscess status.

Characteristic	Abscess		*p* Value	All
	No	Yes		
	*n* (%)	*n* (%)		*n* (%)
Patient type				
Emergency department	20 (76.9)	6 (23.1)	0.92	26 (26.0)
Inpatient	33 (73.3)	12 (26.7)		45 (45.0)
Outpatient	21 (72.4)	8 (27.6)		29 (29.0)
Fever				
No	52 (75.4)	17 (24.6)	0.63	69 (69.00)
Yes	22 (71.0)	9 (29.0)		31 (31.0)
Redness				
No	22 (81.5)	5 (18.5)	0.44	27 (27.0)
Yes	52 (71.2)	21 (28.8)		73 (73.0)
Swelling				
Unknown	1 (100.0)	0 (0)		1 (1.0)
No	37 (75.5)	12 (24.5)	0.82	49 (49.0)
Yes	36 (72.0)	14 (28.0)		50 (50.0)
Focal pain				
No	68 (73.1)	25 (26.9)	0.67	93 (93.0)
Yes	6 (85.7)	1 (14.3)		7 (7.0)
Palpable abnormality				
Unknown	1 (100.0)	0 (0)		1 (1.0)
No	69 (76.7)	21 (23.3)	0.05	90 (90.0)
Yes	4 (44.4)	5 (55.6)		9 (9.0)
History of breast abscess				
No	68 (74.7)	23 (25.3)	0.69	91 (91.0)
Yes	6 (66.7)	3 (33.3)		9 (9.0)
History of breast cancer				
No	10 (90.9)	1 (9.1)	0.28	11 (11.0)
Yes	64 (71.9)	25 (28.1)		89 (89.0)
Smoking status				
Unknown	1 (50.0)	1 (50.0)		2 (2.0)
No	70 (76.1)	22 (23.9)	0.17	92 (92.0)
Yes	3 (50.0)	3 (50.0)		6 (6.0)
Diabetes mellitus				
No	65 (74.7)	22 (25.3)	0.74	87 (87.0)
Yes	9 (69.2)	4 (30.8)		13 (13.0)
Immunocompromised				
No	62 (77.5)	18 (22.5)	0.15	80 (80.0)
Yes	12 (60.0)	8 (40.0)		20 (20.0)
Surgery in preceding 8 weeks				
No	35 (87.5)	5 (12.5)	0.02	40 (40.0)
Yes	39 (65.0)	21 (35.0)		60 (60.0)
After-hours interventional treatment				
No	71 (78.9)	19 (21.1)	0.003	90 (90.0)
Yes	3 (30.0)	7 (70.0)		10 (10.0)
